# Follicle-like tertiary lymphoid structures: A potential biomarker for prognosis and immunotherapy response in patients with laryngeal squamous cell carcinoma

**DOI:** 10.3389/fimmu.2023.1096220

**Published:** 2023-01-27

**Authors:** Haifeng Liang, Zhigang Zhang, Zhong Guan, Shibie Zheng, Jintao Lou, Wei Liu, Qian Cai, Yu Si

**Affiliations:** Department of Otolaryngology- Head and Neck, Sun Yat-sen Memorial Hospital, Sun Yat-sen University, Guangzhou, China

**Keywords:** tertiary lymphoid structures, maturity, laryngeal cancer, immunotherapy, tumor immune microenvironment

## Abstract

**Background:**

The maturity and spatial distribution of tertiary lymphoid structures (TLSs) vary dynamically within and between cancers, leading to a controversial role in cancer. We aimed to develop a simple morphology-based approach to identify the maturity of TLSs in laryngeal squamous cell carcinoma and examine their clinically relevant functional role.

**Methods:**

TLSs were identified based on morphological features *via* hematoxylin and eosin (H&E) staining, and the accuracy was verified by multi-immunohistochemical analysis. The density, maturity, spatial distribution and prognostic value of TLSs were separately analyzed in two human laryngeal cancer cohorts. The TLS profile was linked to RNA-seq data from the TCGA database to perform bioinformatics analysis.

**Results:**

TLSs can be classified as early TLSs (E-TLSs), primary follicle-like TLSs (PFL-TLSs) and secondary follicle-like TLSs (SFL-TLSs). The three types of TLSs showed higher infiltration in the extratumoral region. XCL2 is a vital chemokine in the maturation and infiltration of TLSs. FL-TLS was an independent positive prognostic indicator in laryngeal cancer. The FL-TLS group had more abundant immune cell infiltration and a better response to immunotherapies than the non-FL-TLS group. Functional analysis showed that the non-FL-TLS group was enriched in tumor invasion, metastasis and immunosuppression pathways.

**Conclusion:**

The maturity of TLSs can be accurately classified by H&E staining. FL-TLS is a potential mediator of antitumor immunity in human laryngeal cancer.

## Introduction

The tumor immune microenvironment (TIME) is a spatially structured milieu where immune cells are mostly localized in the center of the tumor, invasive margin, or surrounding stroma (1). Spatial data in conjunction with immunological makeup and cellular conditions can help establish individualized immunological therapy. Three general classes of TIME can be described according to the distribution of immune cells (2): 1) infiltrated-excluded; 2) infiltrated-inflamed; and 3) presence of tertiary lymphoid structures (TLSs), a subclass of the infiltrated-inflamed TIME. Three independent studies published in 2020 showed that B cells and TLSs are important factors influencing the immune checkpoint inhibition response and that TLSs are a potential effective marker for the selection of patients for immune checkpoint inhibitors (ICIs) (3–5).

TLS-TIMEs show histological evidence of lymphocyte aggregates with a similar cellular composition to lymph nodes, including naive and activated CD4+ T cells, CD8+ T cells, B cells, and dendritic cells (DCs). DC-LAMP^+^ DCs and infiltrating lymphocytes in TLSs are closely associated with tumor regression (6). However, the composition of TLSs changes dynamically with the extent and balance of immune cell subsets within and between tumors (7). According to their composition, TLSs can be classified as follows: 1) Early TLSs (E-TLSs) are distinguished by lymphocytic aggregates that lack a DC scaffold and vascularization. 2) Primary follicle-like TLSs (PFL-TLSs), also known as immature TLSs, are primarily comprised of T-cell and B-cell zones with follicular dendritic cells (FDCs) but no germinal centers (GCs). 3) Secondary follicle-like TLSs (SFL-TLSs), also known as mature TLSs, are comprised of lymphatic vessels, isolated T-cell zones, and a B-cell follicle with membranes (8). According to prognostic studies of TLSs in human cancer, TLSs are typically but not always associated with a positive outcome. For example, in cohorts of renal cell, lung, and colon cancers, TLS formation was associated with a better overall survival or magnitude of response to immunotherapy (9) (10). A study showed that TLS formation in nontumoral regions of liver cancer is associated with a poorer prognosis (11). However, another study of liver cancer showed that TLS formation with GCs in the extratumoral region was associated with an improved prognostic outcome, which was further improved if both intratumoral and extratumoral TLSs were formed (12). Therefore, it is anticipated that the creation of standardized methods to assess TLS density, composition, and location would further illuminate its predictive and prognostic relevance in a variety of cancers. TLSs need to be more fully characterized based on composition, maturity, and functional aspects.

Histopathology has unique advantages in analyzing the microenvironment of solid tumors in light of its ability to depict the tumor spatial environment and structural features of TLSs and how they interact with each other (13). These spatial data can help accurately identify the localization and structural characteristics of TLSs and yield more powerful predictions than basic TLS counts.

Laryngeal cancer accounted for 184,615 new cases in 2020 worldwide, representing one-third of all head and neck cancers, with 99,840 associated deaths (14). Unfortunately, despite the laryngeal cancer incidence decreasing overall over the past 40 years, the 5-year survival rate has not changed appreciably (15). This highlights the need for further research and innovation in the treatment of laryngeal cancer. Immunotherapy, as a novel treatment, can eliminate cancer cells by enhancing immune system activity and has been widely used in advanced laryngeal cancer treatment (16). The immune system is closely related to the development and progression of laryngeal cancer. Effective antitumor responses in laryngeal cancer stem mainly from adaptive immunity maintained by T cells and B cells, as well as TLSs that can coordinate endogenous antitumor immune responses (17). The current study partially investigated the prognostic role of TLSs in head and neck squamous cell carcinoma (18), but research on the role of TLS maturity and spatial distribution in patient prognosis remains lacking. In addition, the prognosis of patients with different sites of head and neck squamous cell carcinoma (such as common hypopharyngeal carcinoma and laryngeal carcinoma) varies greatly. Thus, we focused on laryngeal cancer rather than head and neck squamous cell carcinoma as the study object in the survival analysis. Small-sample studies have revealed that the formation of TLSs is connected to longer disease-free survival (DFS) and a lower recurrence rate (RR) in laryngeal cancer (19, 20), but there is still a lack of studies on the effect of TLS maturity stage and localization on the prognosis of laryngeal cancer.

In our study, we first identified the TLS maturity stage based on morphological features *via* hematoxylin and eosin (H&E) staining and verified the accuracy by multi-immunohistochemical analysis, followed by analyses of the spatial distribution and prognostic significance of different TLS maturity stages *in situ* in tissues. In addition, we used The Cancer Genome Atlas (TCGA) database to examine the factors that contribute to the different prognoses of different TLS maturities from a variety of perspectives, such as the composition of the immune microenvironment, prediction of immunotherapy response, and enrichment of gene functional pathways.

## Method

### Study design and clinical sample collection

The purpose of our study was to investigate the morphological differences in TLSs with different maturities in human tumor tissues and their associated mechanisms affecting prognosis. To this end, we collected tumor tissue sections and clinical information from patients who underwent laryngeal squamous cell carcinoma (LSCC) resection surgery at Sun Yat-sen Memorial Hospital, Sun Yat-sen University, from 2012 to 2020. All tumor samples were taken with the informed consent of the patients. The patients were included based on the following criteria: pathological diagnosis of squamous cell carcinoma, availability and good quality of the obtained tissue section, complete clinical information and follow-up data, and no prior anticancer therapy. Ultimately, tissue sections and clinical data from 105 patients were included in our observational study (test cohort, **Table 1**). Survival data were collected retrospectively, and overall survival (OS) was selected as the primary endpoint. Survival analysis was performed for patients who were followed up for at least 24 months (mean 58 months).

**Table 1:** Clinical characteristics for all patient population.

Characteristic	TCGA Cohort–NO(%)	Test Cohort–NO(%)
**All patients**	82	105
Sex		
**Female**	11 (13)	5 (4)
**Male**	71 (87)	100 (96)
**Age**		
<60	31 (39)	47 (45)
≥60	51 (61)	58 (55)
T stage		
1-2	10 (11)	46 (44)
3-4	68 (85)	59 (56)
X	4 (4)	0 (0)
**N stage**		
0	37 (45)	81 (77)
X	45 (55)	24 (23)
TLS Group		
SFL-TLS	19 (23)	31 (30)
PFL-TLS	24 (29)	31 (30)
E-TLS	34 (41)	33 (31)
Non-TLS	5 (6)	10 (9)
TLS intratumoral infiltration		
Yes	41 (50)	55 (52)
No	41 (50)	50 (48)
Survival rate		
3 year	61.8%	83.2%
5 year	55.2%	74.6%

### Multiplex immunohistochemical staining and image acquisition

For multiplex immunohistochemical (m-IHC) staining, primary antibodies were sequentially applied to deparaffinized sections of formalin-fixed paraffin-embedded (FFPE) tumor samples for three rounds of iterative immunostaining. At the beginning of each round, heated antigen retrieval was performed with EDTA (Solarbio, 50x, pH 8.0) for 20 min, and a blocking step was performed using 20% normal goat serum for 10 min. Following the application of primary antibodies, HRP-labeled secondary antibodies of the mouse/rabbit mixed type (Akoya) and fluorophore dye (Akoya) were added. Finally, DAPI (PerkinElmer, FP1490) was used to counterstain the nuclei after three rounds of immunostaining. To guarantee contextual specificity, all antibodies were initially tuned using IHC on tonsillar tissues (**Supplementary Figure S1**). The signal-to-noise ratio was then optimized by repeatedly modifying the fluorophore pairings and concentrations after the antibodies had been tested by immunohistochemistry. The primary antibodies used for immunostaining and their paired fluorophores are shown in **Supplementary Table S1**.

m-IHC slides of the sections were scanned on a Vectra Polaris Automated Quantitative Pathology Imaging System (Akoya). Whole fluorescence signals were imaged as multispectral high-power fields (20x), and the resulting spectra were unmixed by Inform^®^ (version 2.50, Akoya).

### Image analysis

All images were manually histologically analyzed and spatially annotated by a sample analyst blinded to the clinical information *via* the digital pathology image analysis software QuPath (version 0.2.3). Intratumoral and extratumoral areas were defined according to the tumor tissue invasion boundary, where peritumoral areas were defined as areas (mm^2^) within 2 mm outside of the tumor invasion boundary. The density of TLSs was defined as the ratio of the TLS area to the intratumoral or peritumoral area.

### TCGA raw data

The TCGA database (https://portal.gdc.cancer.gov/, version 33.0, March 22, 2022) was used to download the RNA sequencing (RNA-seq) data (**Supplementary Data 1**), clinicopathological data (**Supplementary Data 2**) and pathological diagnostic slides of 459 head and neck squamous cell carcinoma (HNSC) patients. A total of 82 LSCC patients with complete follow-up records and H&E pathological diagnosis section data were finally selected (TCGA cohort, **Table 1**). HTSeq-FPKM data from 82 samples were used for CIBERSORT, MCP-Counter, and xCell analyses. HTSeq-Counts data were used for differential analysis.

### Analysis of immunological profiles and ICI treatment response in different TLS maturity subgroups

To identify immunological profiles in TCGA samples, their HTSeq-FPKM data were uploaded into CIBERSORT (21), MCP-Counter (22), and xCell (23) to obtain the variations in immune cell infiltration among various TLS maturity groupings.

The |RGP| score is an immunotherapy-related prognostic index constructed based on the gene expression of SFRP4, CPXM1, and COL5A1 in head and neck cancer samples (24). |RGP| score = (− 0.18) * gene expression value of SFRP4 + 0.29 * gene expression value of CPXM1 + (− 0.23) * gene expression value of COL5A1.

The limma package was loaded on R software (version 4.0.4) for screening differentially expressed genes. Kyoto Encyclopedia of Genes and Genomes (KEGG) pathway analysis between the follicular TLS (FL-TLS group) and the non-follicular TLS (nFL-TLS) group was performed using GSEA software (version 4.1.0).

### Statistical methods

The agreement of the TLS maturity typing results between fluorescence-stained images and H&E-stained images was tested by quadratic weighted kappa. The Mann−Whitney test was used to compare the infiltration of different immune cells and |RGP| scores between groups forming FL-TLS and nFL-TLS structures. The Kruskal−Wallis test was used to compare the infiltration of tumor-infiltrating immune cells and |RGP| scores between groups with different TLS maturity types. For the TCGA cohort and test cohort, OS was defined as the time from the date of surgery to the date of death or the final follow-up (the TCGA cohort survival data were last updated in March 2022, and the test cohort’s last follow-up data were collected in May 2022). Kaplan−Meier survival curves for OS were drawn, and statistical significance (defined as P<0.05) was assessed using log-rank regression analysis. Univariate and multivariate Cox regression analyses were used to identify independent variables influencing OS. The above statistical analyses were performed with SPSS 26.0 and R software (version 4.0.4). Visualization of the statistical findings in this article was performed by R software (version 4.0.4) and GraphPad Prism (version 8.0).

## Results

### Definition of TLS maturity states in human LSCC

**Figure 1:**
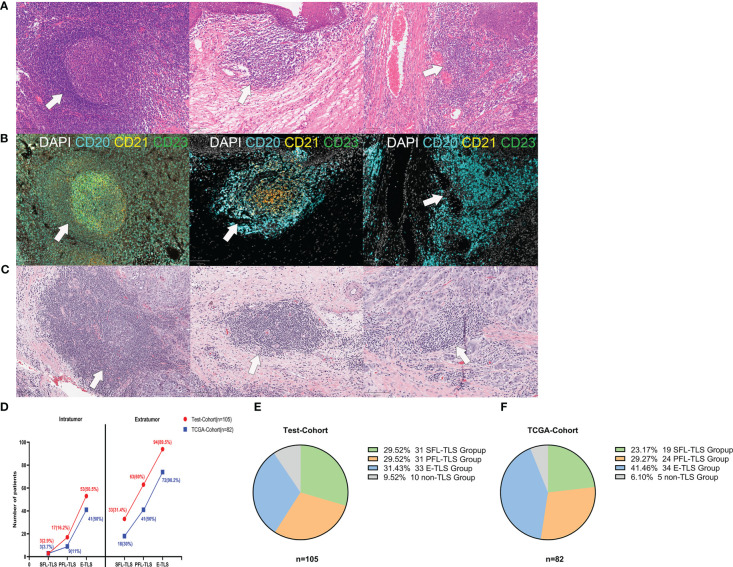
Morphological characteristics of TLSs at different stages of maturity. **(A)** Representative images of TLSs at different maturity stages in the test cohort. (1) Secondary follicle-like TLSs (SFL-TLSs, left image); (2) primary follicle-like TLSs (PFL-TLSs, middle image); (3) early TLSs (E-TLSs, right image). **(B)** (1) SFL-TLS, CD21 and CD23 signal expression in dense CD20+ lymphocyte clusters; (2) PFL-TLS, CD21 signal expression in dense CD20+ lymphocyte clusters, but no CD23 signal expression; (3) E-TLS, CD20+ lymphocyte clusters lacking CD21 and CD23 expression. **(C)** Representative images of TLSs at different maturity stages in the TCGA cohort. **(D)** Frequency of TLSs at different maturity stages in intratumoral versus extratumoral regions. **(E)** Grouping of patients according to TLS maturity in the test cohort. **(F)** Grouping of patients according to TLS maturity in the TCGA cohort.

It is well known that the spatial context of immune cells is critical to the genesis of cancer. To detect the spatial heterogeneity of tumors at the cellular level, pathological evaluation is essential. To identify the presence, location and maturity states of TLSs in LSCC, LSCC patients were enrolled as the test cohort, and their samples were stained with H&E (n=105) and m-IHC (n=40) (**Figures 1A, B**). TLSs were categorized by two independent pathologists according to their morphological differences as follows: (1) SFL-TLSs were identified as dense clusters of lymphocytes forming oval-like follicular structures with well-defined GC areas, and CD21+ and CD23+ cells were present in CD20+ lymphocyte clusters; (2) PFL-TLSs were identified as dense clusters of lymphocytes forming oval-like follicular structures without GCs, and only CD21+ cells were present in CD20+ lymphocyte clusters; and (3) E-TLSs were identified as amorphous, dense lymphocyte clusters (greater than 10,000 μm^2^) that did not form follicular structures, with CD20+ lymphocyte clusters lacking CD21+ and CD23+ cells (**Supplementary Figure S2**). Subsequently, we performed the same TLS maturity stage division on H&E-stained sections of laryngeal carcinoma from the TCGA database (**Figure 1C**). To more intuitively reflect the differences in TLS maturity in patient tissues, we divided the patients into SFL-TLS, PFL-TLS, E-TLS, and non-TLS groups according to the highest maturity stage of TLSs in the pathological sections of each patient. The accuracy of patient grouping was confirmed by randomly selecting 40 pathological sections of LSCC from the 105 patients in the test cohort and determining their TLS maturity grouping using 4-color m-IHC. The weight kappa agreement test was performed between the maturity staging results of TLSs based on H&E staining and those based on immunohistochemical staining. The results suggested strong agreement between them (**Supplementary Table S2**, Kappa 0.810, P<0.001), indicating that the TLS maturity stage grouping could be accurately identified based on H&E staining.

TLSs of varying maturity stages can be identified in both intratumoral regions and extratumoral regions. In both regions, E-TLS was the most frequent phenotype, followed by PFL-TLS and SFL-TLS. Interestingly, significantly more TLSs were present in the extratumoral regions than in the intratumoral regions (**Figure 1D**). Of 105 patients in the test cohort, the E-TLS group was the most common (31.43%), followed by the SFL-TLS and PFL-TLS groups (29.59%, each). However, there were no TLSs present in ten patients (9.52%) (**Figure 1E**). Among 82 laryngeal cancer samples in the TCGA cohort, 19 samples were identified as the SFL-TLS group (23.17%), 24 as the PFL-TLS group (27.27%), 34 as the E-TLS group (41.46%), and 5 as the non-TLS group (6.10%) (**Figure 1F**).

### Spatial distribution and clinical relevance of TLSs

**Figure 2:**
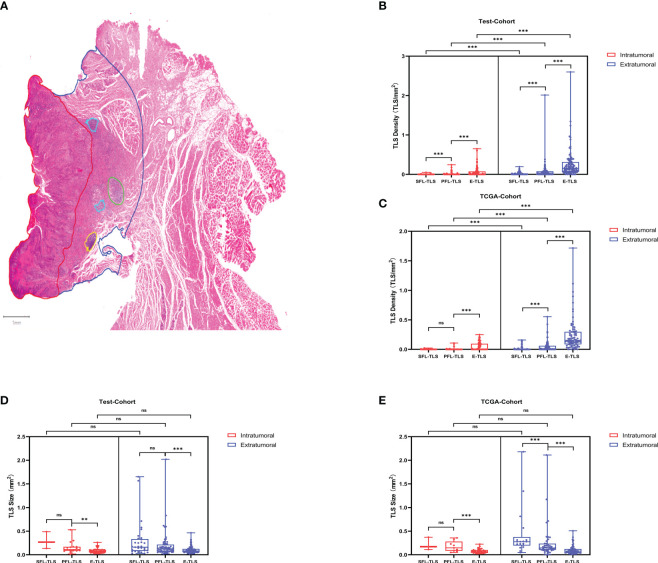
TLS maturity, density and spatial distribution. **(A)** Detection of TLSs at different stages of maturity in LSCC diagnostic H&E sections. Spatial annotation: Intratumoral and extratumoral areas were defined according to the tumor invasion boundary, where extratumoral stromal areas were defined as the areas within 2 mm outside of the tumor invasion boundary. The density of TLSs was defined as the ratio of the TLS number to the intratumoral area/extratumoral area. **(B)** Density differences between TLSs at different maturity stages in the test cohort. **(C)** Density differences between TLSs at different maturity stages in the TCGA cohort. **(D)** Average size differences of TLSs at different maturity stages in the test cohort. **(E)** Average size differences in TLSs at different maturity stages in the TCGA cohort. (ns: not significance,***P<0.001, upper whisker represents the maximum).

To investigate the spatial distribution of TLSs, we compared the density and average size of TLSs in the intratumoral and extratumoral regions (**Figure 2A**). The test cohort and TCGA cohort consistently showed that E-TLSs had a significantly greater density than SFL-TLSs and PFL-TLSs and that the density of PFL-TLSs was significantly higher than that of SFL-TLSs. Moreover, the density of TLSs was significantly higher in the extratumoral regions than in the intratumoral regions (**Figures 2B, C**). In contrast, the average size of PFL-TLSs was significantly larger than that of E-TLSs in both cohorts, whereas the average size of SFL-TLSs was significantly larger than that of PFL-TLSs only in the TCGA cohort. Notably, TLS size in the intratumoural region was not significantly different from that in the intratumoural region (**Figures 2D, E**). In addition, we also analyzed the relationships of TLS maturity status with clinical information in the test cohort and TCGA cohort. There was a slight correlation between age at onset and TLS maturity in the TCGA cohort but no correlation between TLS maturity and age at onset in the test cohort. Neither tumor T stage nor N stage showed a significant correlation with TLS maturity in either cohort (**Supplementary Table S3**). Based on the above analysis, we found that in higher maturity stages, the density of TLSs was much lower, whereas the average size of TLSs was significantly larger in the higher maturity stage, indicating that TLS formation may be a process from the lower maturity grade to the higher maturity grade, and this process seems to be independent of the degree of tumor progression. In addition, TLSs at any stage of maturity have difficulty infiltrating tumors.

### Follicular TLS formation is associated with a better prognosis

**Figure 3:**
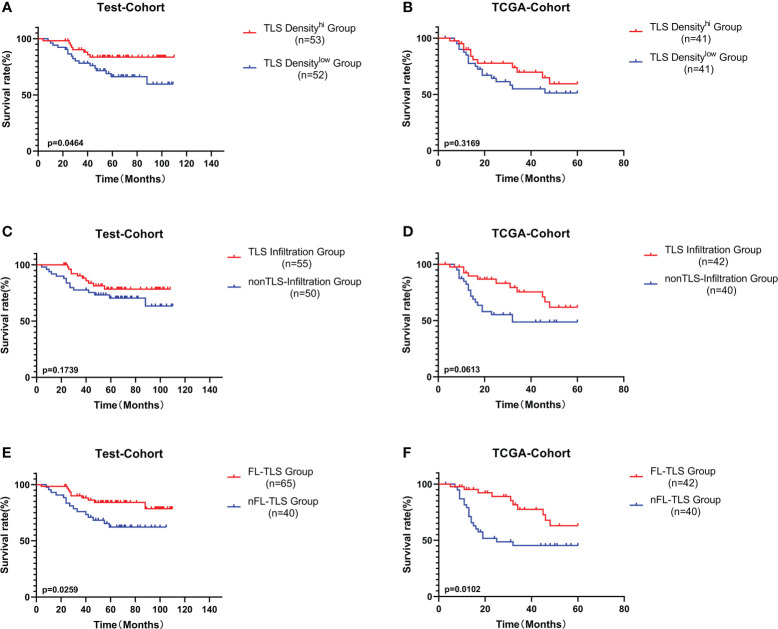
Prognostic analysis of different TLS-related features. **(A)** Patients were divided into the TLS density-high group and TLS density-low group by the median cutoff value. K-M survival analysis of TLS density subgroups in the test cohort. **(B)** K-M survival analysis of TLS density subgroups in the TCGA cohort. **(C)** K-M survival analysis between the TLS infiltration group and the non-TLS infiltration group in the test cohort. **(D)** K-M survival analysis between the TLS infiltration group and the non-TLS infiltration group in the TCGA cohort. **(E)** K-M survival analysis between the FL-TLS group and the nFL group in the test cohort. **(F)** K-M survival analysis between the FL-TLS group and the nFL group in the TCGA cohort.

Subsequently, we explored the prognostic value of TLS-related features. Similar to other studies, higher TLS density was associated with a better prognosis in LSCC in both the test cohort and the TCGA cohort but was only significant in the test cohort (**Figures 3A, B**). Furthermore, we analyzed the influence of the spatial distribution of TLSs on survival. According to the infiltration of TLSs in intratumoral regions, the patients were divided into two groups: the TLS infiltration group and the non-TLS infiltration group. The TLS infiltration group showed a better prognostic trend, but the difference was not significant (**Figures 3C, D**). In both the test cohort and TCGA cohort, patients with FL-TLS formation, i.e., the SFL-TLS and PFL-TLS groups, had a significantly better OS than patients with nFL-TLS, i.e., the E-TLS and non-TLS groups (**Figures 3E, F**). There was no significant difference in prognosis between the SFL-TLS and PFL-TLS groups or between the E-TLS and non-TLS groups (**Supplementary Figure S3A**). In addition, we found that the formation of FL-TLS in the extratumoral region was associated with a significantly better prognosis, whereas patients with FL-TLS formation in the intratumoral region all had a good prognosis, but statistical significance could not be reflected due to sample size constraints (**Supplementary Figures S3B, C**).

Next, we conducted univariate and multivariate Cox regression analyses of other common tumor-related clinical parameters. Among the tumor-related parameters, FL-TLS formation was the only independent predictor of OS in multivariate Cox regression analyses in both cohorts (**Supplementary Figure S4**). In conclusion, FL-TLS formation is associated with a better prognosis in LSCC, and the presence or absence of FL-TLS formation in LSCC as an independent factor affecting the prognosis of laryngeal carcinoma can be used as a meaningful prognostic indicator in laryngeal carcinoma.

### FL-TLS is a potential mediator of antitumor immunity

**Figure 4:**
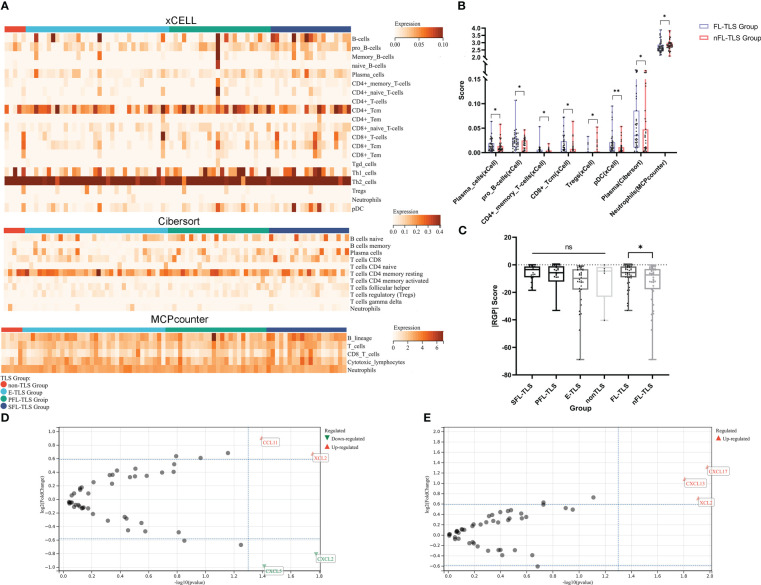
Immune cell infiltration landscape and chemokine gene features in LCSS. **(A)** Heatmap for the immune cell infiltration landscape based on the xCell, CIBERSORT, and MCP-Counter algorithms among different TLS maturity groupings (only items with significant differences, B-cell lines and T-cell lines are displayed). **(B)** Heatmap of the immune cell infiltration landscape between the FL-TLS and nFL-TLS groups (only items with significant differences). **(C)** |RGP| score across different TLS maturity groupings. **(D)** Chemokine genes differentially expressed in the FL-TLS group compared with the nFL-TLS group. **(E)** Chemokine genes differentially expressed in the TLS infiltration group compared with the non-TLS infiltration group. (ns: not significance, *P<0.05, **P<0.01,upper whisker represents the maximum).

To comprehend the potential causes underlying the prognostic advantage of FL-TLS formation, we comparatively analyzed the immune microenvironment composition of patients in the TCGA cohort with FL-TLS versus nFL-TLS. The immune cells in the different TLS maturity groups were assessed by the xCell, CIBERSORT, and MCP-Counter algorithms (**Figure 4A**, **Supplementary Data 3**). The results of the above algorithms showed that antitumor-related immune cells such as plasma cells, pro-B cells, CD4+ memory T cells, CD8+ Tcm cells, and pDCs were significantly more abundant in the FL-TLS group, while immunosuppressive-related cells such as Treg cells and neutrophils were significantly more abundant in the nFL-TLS group (**Figure 4B**). The clinical efficacy of immunotherapy for the FL-TLS and nFL-TLS groups was then evaluated by |RGP|. An active immune response and a less aggressive tumor phenotype are indicated by a higher |RPG| prediction score, which suggests that patients would probably benefit from ICI therapy. According to our findings, the |RPG| score in the FL-TLS group was significantly higher than that in the nFL-TLS group (**Figure 4C**). Overall, the formation of FL-TLSs may be associated with more abundant immune infiltration and a better ICB treatment response.

### The expression of multiple chemokine genes in tumors is significantly correlated with the presence and infiltration of TLSs

To determine the elements that influence the maturity of TLSs, we analyzed differences in the expression of common chemokine genes between the FL-TLS group and the nFL-TLS group (**Supplementary Table S4**). We found that the CCL1 and XCL2 genes were significantly upregulated, whereas the CXCL5 and CXCL2 genes were significantly downregulated in the FL-TLS group (**Figure 4D**). In addition, we found significant upregulation of the CXCL13, XCL2, and CXCL17 genes in samples with TLS infiltration in the tumor (**Figure 4E**). Significantly associated with TLS maturity and infiltration, XCL2 is a chemokine produced by NK cells that stimulates DC recruitment into the tumor microenvironment. These results therefore indicated that XCL2 may be a vital chemokine in the maturity and infiltration of TLSs in human LSCC.

### Enriched gene sets in different TLS states

**Figure 5:**
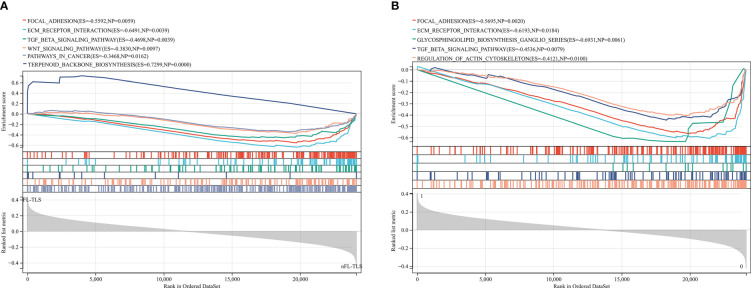
Gene enrichment analysis for different TLS maturity stages and spatial distributions. **(A)** Enrichment of gene sets in the nFL-TLS group. **(B)** Enrichment of gene sets in the TLS infiltration group.

To identify the gene differences in TLSs with different states, including location, maturity, and function, KEGG analysis was performed using GSEA to identify the gene sets enriched between the FL-TLS and nFL-TLS groups. The gene sets enriched in the nFL-TLS group were tumor invasion, metastasis and immunosuppression pathways, including ECM receptor interactions, focal adhesion, the TGF-β signaling pathway and the WNT signaling pathway (**Figure 5A**; P< 0.05, FDR < 0.25). The same analytical method was performed to determine the gene sets enriched between the groups with and without TLS infiltration in the tumor. The gene sets of the group without TLS infiltration in the tumor were enriched in tumor immune escape pathways, including the glycosphingolipid biosynthesis pathway, regulation of action cytoskeleton pathway, and TGF-β signaling pathway (**Figure 5B**; P< 0.05, FDR < 0.25). To gain further biological insight, we performed mutational analysis between the above groups. Missense variations were the most common mutation type, and we list the top 20 genes with their mutation rates in each subgroup in the supplemental material (**Supplementary Figure S5**).

## Discussion

The TIME and clinical outcomes of cancer patients are closely correlated (25). TLS, a unique structure in the TIME, is considered a critical factor in anticancer immune responses (2, 26). The formation of TLSs may vary between cancer types, and there is currently no widespread agreement on the definition and measurement of TLSs in human cancer. In existing datasets, it is difficult to directly compare TLSs because the markers used to identify TLSs are not the same (27).

In our study, we provided a morphology-based method to identify stages of TLS maturity in H&E-stained sections and validated the accuracy of this method by multicolor immunohistochemistry. Then, for the first time, we assessed the maturity characteristics of TLSs in two LSCC cohorts. Analyses of both cohorts consistently demonstrated the presence of intratumoral and peritumoral TLSs of varying maturity in LSCC, with mature TLSs and intratumoral TLSs occurring significantly less frequently. In addition, our study suggests that stages of TLS maturity can be precisely measured in diagnostic H&E sections, making them suitable for introduction as relevant prognostic criteria in routine pathology. This study may help to establish a clinical standard procedure for analyzing TLS maturity, density, and spatial distribution in human cancers in the future.

Numerous studies have shown that TLS density is generally associated with a better prognosis, and TLS density is an independent prognostic marker in lung (28), colorectal (29), breast (30), and pancreatic cancers (31). Our findings similarly suggest that the density of TLSs is correlated with the prognosis of LSCC but does not serve as an independent prognostic indicator of LSCC. There is still a lack of studies on the relationship between prognosis and TLS maturity. Our two cohort studies showed that the presence of TLSs at different stages of maturity is associated with different prognoses. More accurately, the formation of FL-TLSs represents a better prognosis, suggesting that FDCs in TLSs may play an important antitumor role. FDCs are immune system cells that can be found in B-cell follicles, and they have the extraordinary capacity to store intact antigen for extended periods of time (32). With the help of FDCs, B cells can acquire antigens that far exceed those initially infected or damaged. In this process, FDCs also play a role in screening B cells because only B cells with enough high-affinity receptors can successfully receive enough antigens from FDCs (33). Interestingly, we found that the formation of SFL-TLS with GCs did not show a good prognosis in LSCC, as expected. It has been shown that GCs in tumor-associated TLSs exhibit weaker proliferative activity and decreased Ki67 and BCL6 expression compared to GCs in secondary lymphoid organs (13). This suggests that further studies on the functional status of GCs in addition to focusing on their formation are still needed.

Next, we explored why FL-TLS formation is associated with a better prognosis. We used the CIBERSORT, xCell, and MCP-Counter algorithms to analyze the link between FL-TLS formation and immune cell infiltration. W0hile plasma cells, cytotoxic CD8+ T cells, and CD4+ T cells were more enriched in the FL-TLS subgroup, Treg cells and neutrophils were more common in the nFL-TLS subgroup. Numerous studies have demonstrated the intensive infiltration of plasma cells (3, 34) and CD8+ cells (35), indicating a favorable prognosis. CD4+ memory T cells are a subset of tumor-infiltrating T cells capable of continuous immune surveillance to provide long-term immunity (36). On the other hand, our previous studies showed that neutrophils in tumor tissue interact with T cells and inhibit their effector function, thereby inhibiting T-cell antitumor immune responses (37). Therefore, we believe that the formation of FL-TLSs is associated with abundant immune infiltrates and demonstrates an immunologically “hot” profile, which may lead to better efficacy of ICB treatment. To validate the immune-related prognostic role of FL-TLS formation, we investigated the relationship between the FL-TLS group and |RGP|, a known biomarker predictive of immunotherapy response. Here, we found a significant correlation between FL-TLS formation and high |RGP| scores, suggesting that FL-TLS formation may be associated with a better prognosis with immunotherapy.

Through gene enrichment analysis, we found that the gene expression of chemokines seems to be associated with the development and infiltration of TLSs. XCL2 is a chemokine produced by NK cells that stimulates DC recruitment in the tumor microenvironment. Previous studies have identified an NK-DC construct that exerts antitumor effects by presenting tumor antigens and secreting cytokines that regulate T-cell survival and effector function (38, 39). There have been few prior studies on the relationship between NK cells and TLSs, but we found that NK cell-expressed chemokine expression is closely associated with the development and infiltration of TLSs, suggesting a role for NK cells in TLS formation or that similar NK-DC structures may be activated in the anatomical location of TLSs. CXCL13 is mostly generated by perivascular and stromal cells associated with TLSs and may act as a lymphocyte entrance site from the circulation, which is closely associated with the initial formation of TLSs (40). In summary, we propose several chemokines associated with TLS formation versus infiltration, but further determination of their interaction relationship with TLSs at a spatial level is needed.

To further understand the oncological properties of the TLS maturity subgroups, we employed KEGG to perform gene enrichment analysis of the TLS maturity subsets. The TGF-β signaling pathway and WNT signaling pathway were enriched in the nFL-TLS group. During tumor development, the TGF-β signaling pathway achieves immunosuppression by inhibiting the proliferation and function of immunological cells such as T cells. In addition, TGF-β induces the secretion of connective tissue growth factor, endothelin-1, and vascular endothelial growth factor, thus promoting the formation of a tumor metastatic microenvironment (41). The WNT pathway is one of the main signaling cascades of frequent disorders in human cancer. Active WNT signaling is associated with processes that promote tumor development and progression, such as cell proliferation or survival or therapeutic resistance (42). In head and neck squamous cell cancer, higher expression of the TGF-β signaling pathway (43) as well as the WNT signaling pathway (44) has been related to worse patient outcomes, which is consistent with the results of our survival analysis between the FL-TLS group and the nFL-TLS group.

**Figure 6:**
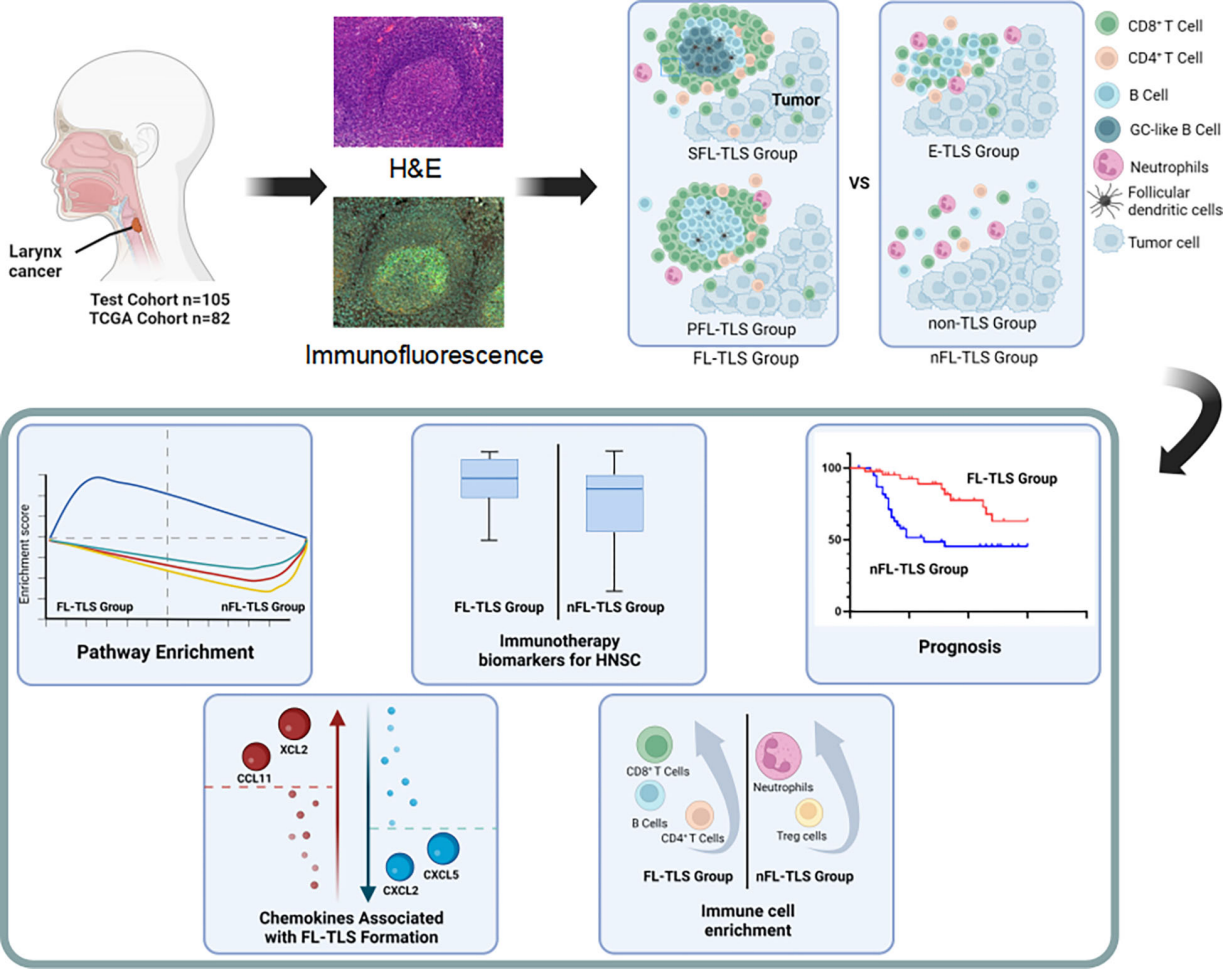
Figure abstract for the comprehensive characterization of the nFL-TLS group in HNSCC.

FL-TLS is a promising prognostic biomarker in LSCC. In addition, the formation of FL-TLSs showed a potential correlation with tumor immune characteristics and the response to immunotherapy (**Figure 6**). However, further clinical studies are needed to validate FL-TLS as a prognostic indicator of the immune response.

## Conclusion

In conclusion, we evaluated immune infiltration in patients with LSCC at the orthotopic tissue level of the tumor. Intratumoral and peritumoral TLSs at different stages of maturity are present in human laryngeal cancer, and the formation of FL-TLSs is associated with a better prognosis. Our study differs from other TLS-related studies in two important ways. First, we identified TLS maturity stages in pathologically stained sections of H&E based on a simple and accurate method. Additionally, for the first time, we linked TLS features in pathological sections to RNA-seq data in the TCGA database and analyzed the reasons why the formation of FL-TLS is associated with a better prognosis.

## Data availability statement

The original contributions presented in the study are included in the article/**Supplementary Material**. Further inquiries can be directed to the corresponding authors.

## Ethics statement

The studies involving human participants were reviewed and approved by Institutional Ethics Committee of Sun Yat-Sen Memorial Hospital of Sun Yat-Sen University (approved number: 2022-KY-064). The patients/participants provided their written informed consent to participate in this study.

## Author contributions

HL, SZ, JL, WL performed the experiments and data analysis. YS, QC, ZZ and ZG were involved in data analysis and technical support. QC provided the study materials. FL, ZZ, QC, and YS designed the experiment, interpreted the data, and wrote the manuscript. All authors contributed to the article and approved the submitted version.

## Supplementary material

Supplementary Figure 1 IHC validation of antibodies on tonsillar tissues.

Supplementary Figure 2 Single spectrum (480, 520, 620) images for each of the individual markers.

Supplementary Figure 3 Prognostic analysis of different TLS maturity stages and spatial locations. **(A)** K-M survival analysis of different TLS maturity subgroups in the test cohort and TCGA cohort. **(B)** K-M survival analysis of the FL-TLS group and nFL TLS group in the intratumoural region in the test cohort and TCGA cohort. **(C)** K-M survival analysis of the FL-TLS group and nFL TLS group in the extratumoral region in the test cohort and TCGA cohort.

Supplementary Figure 4 Prognostic analysis of clinicopathologic factors and different TLS factors. Univariate Cox analyses of common clinicopathologic factors, TLS density, different TLS maturity groupings (FL-TLS group versus nFL-TLS group), and TLS invasion in tumors and multivariate Cox analysis of all univariate Cox analysis items.

Supplementary Figure 5 Significantly mutated genes in the mutant samples. Subtype 0: groups with TLS infiltration in tumors; Subtype 1: groups without TLS infiltration in tumors.
