# Supplementary figures and images for: Follicle-like tertiary lymphoid structures: A potential biomarker for prognosis and immunotherapy response in patients with laryngeal squamous cell carcinoma
Source: Front Immunol. 2023 Jan 27;14:1096220. doi: 10.3389/fimmu.2023.1096220 (PMC9912937; doi:10.3389/fimmu.2023.1096220)

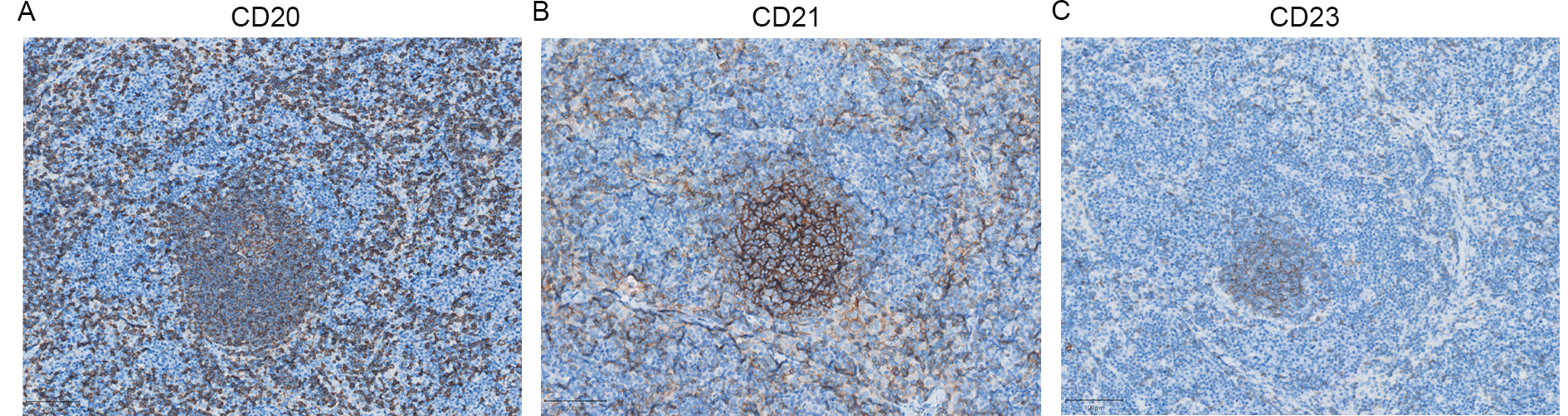

Supplement: Supplementary Figure 1 — IHC validation of antibodies on tonsillar tissues. [file Image_1.tif]

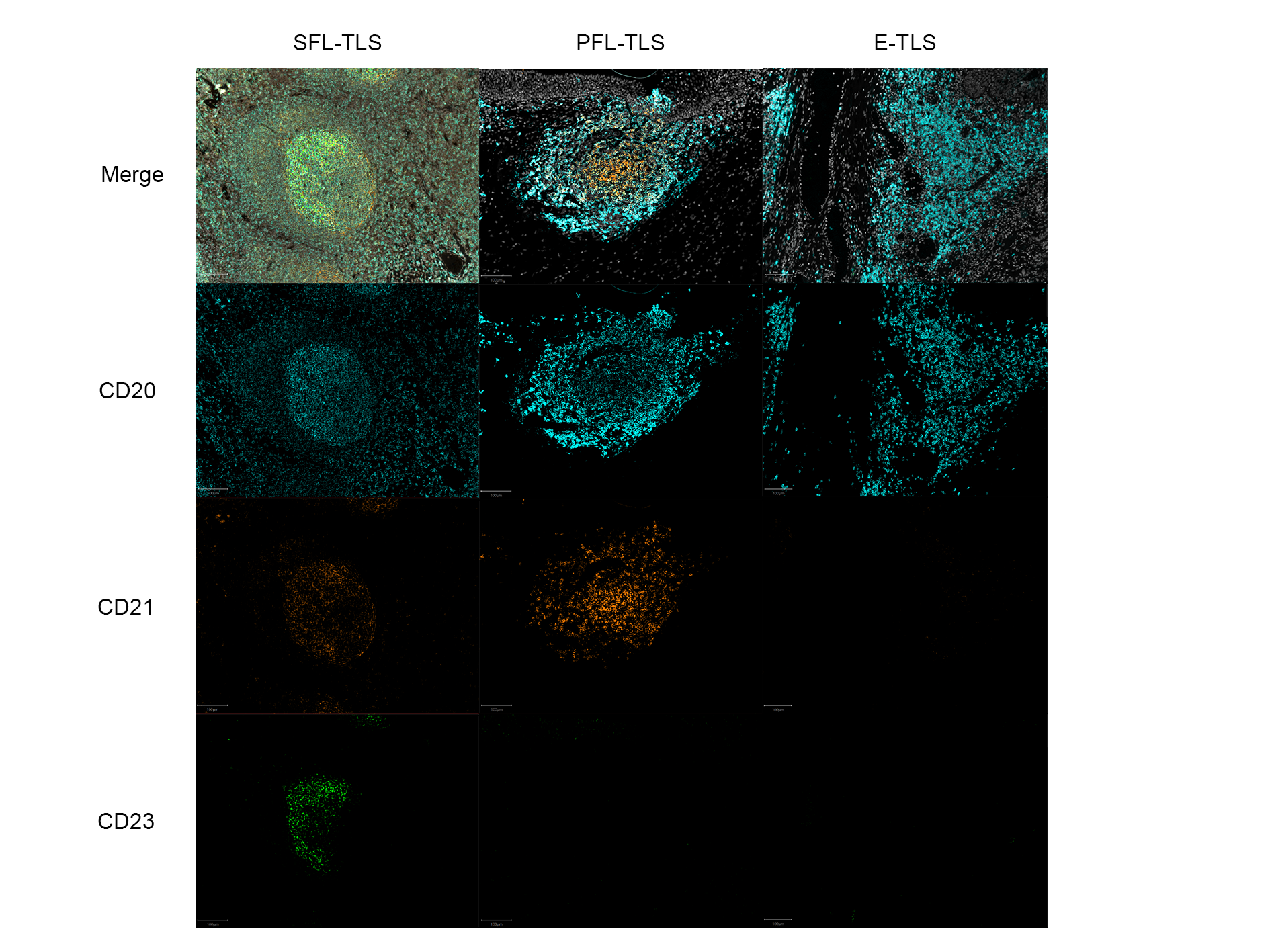

Supplement: Supplementary Figure 2 — Single spectrum (480, 520, 620) images for each of the individual markers. [file Image_2.tif]

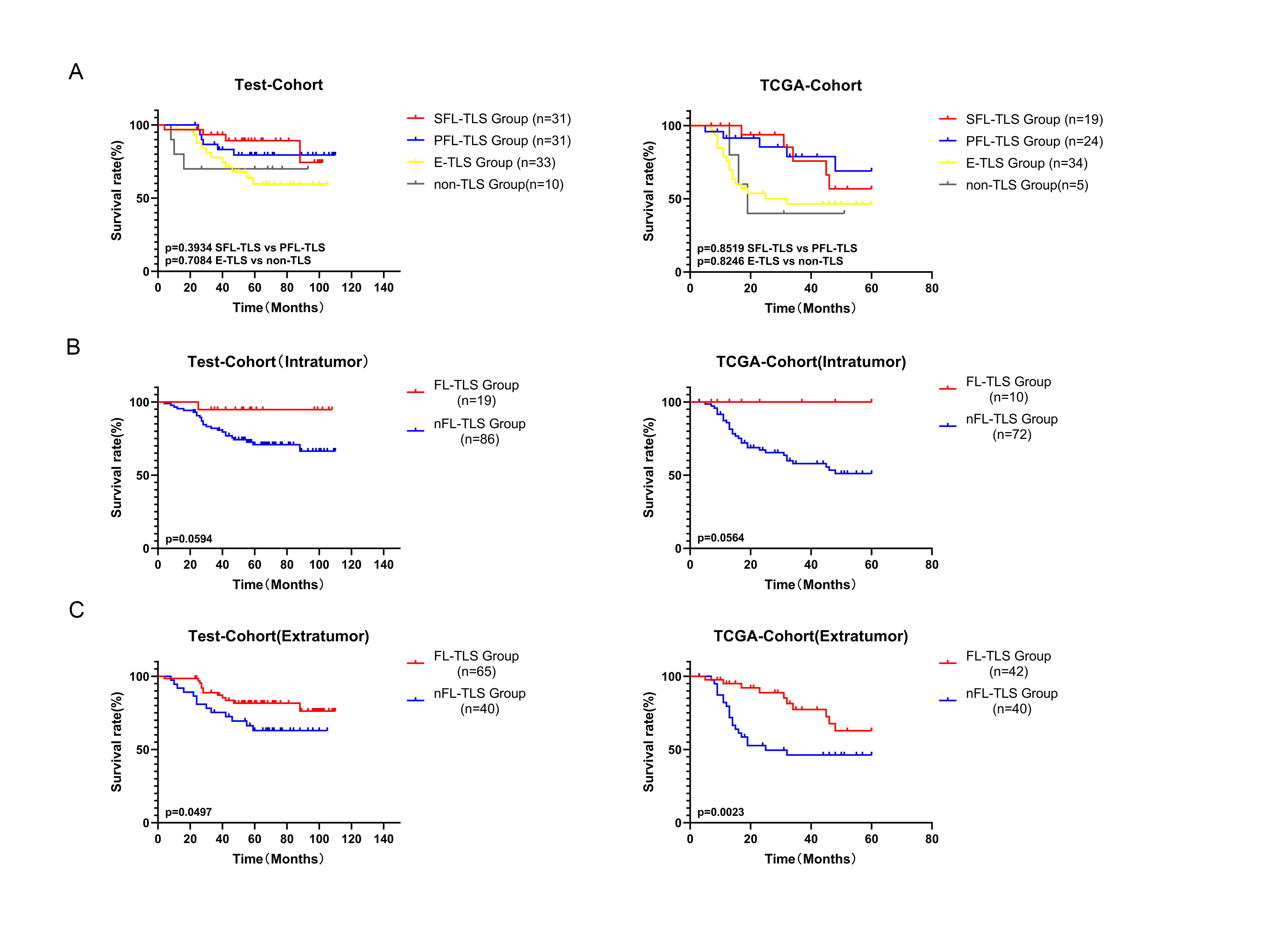

Supplement: Supplementary Figure 3 — Prognostic analysis of different TLS maturity stages and spatial locations. (A) K-M survival analysis of different TLS maturity subgroups in the test cohort and TCGA cohort. (B) K-M survival analysis of the FL-TLS group and nFL TLS group in the intratumoural region in the test cohort and TCGA cohort. (C) K-M survival analysis of the FL-TLS group and nFL TLS group in the extratumoral region in the test cohort and TCGA cohort. [file Image_3.tif]

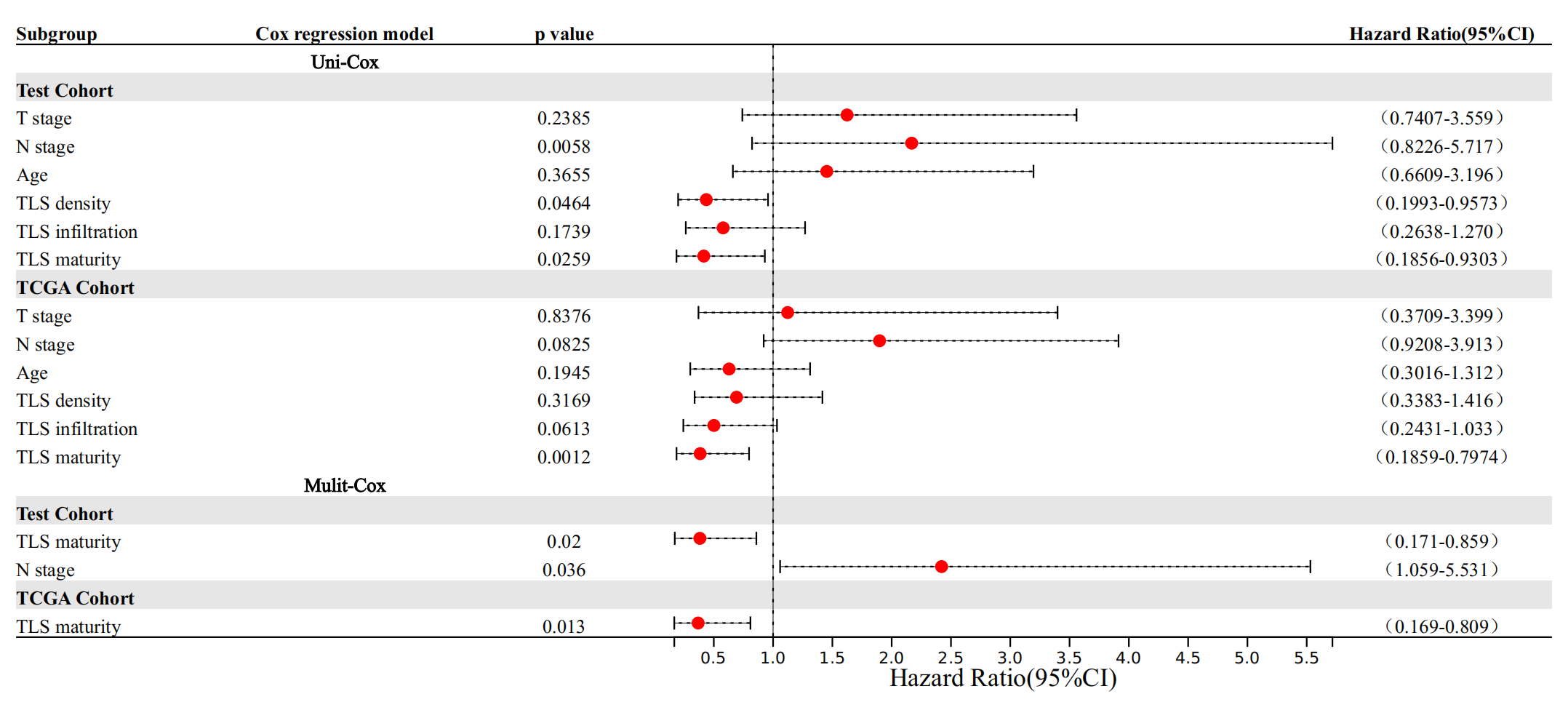

Supplement: Supplementary Figure 4 — Prognostic analysis of clinicopathologic factors and different TLS factors. Univariate Cox analyses of common clinicopathologic factors, TLS density, different TLS maturity groupings (FL-TLS group versus nFL-TLS group), and TLS invasion in tumors and multivariate Cox analysis of all univariate Cox analysis items. [file Image_4.tif]

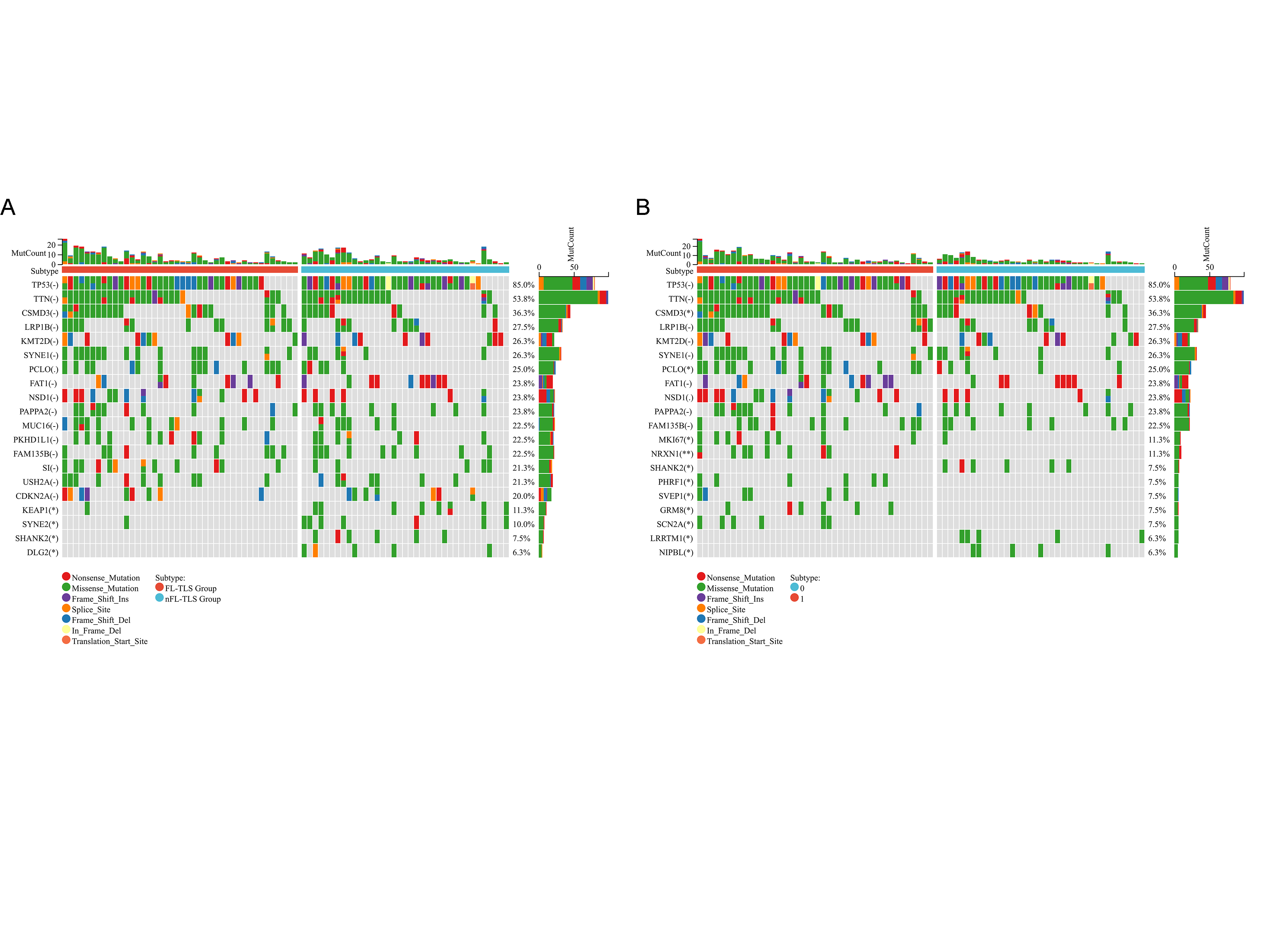

Supplement: Supplementary Figure 5 — Significantly mutated genes in the mutant samples. Subtype 0: groups with TLS infiltration in tumors; Subtype 1: groups without TLS infiltration in tumors. [file Image_5.tif]
