# Supplementary material for: Follicle-like tertiary lymphoid structures: A potential biomarker for prognosis and immunotherapy response in patients with laryngeal squamous cell carcinoma
Source: Front Immunol. 2023 Jan 27;14:1096220. doi: 10.3389/fimmu.2023.1096220 (PMC9912937; doi:10.3389/fimmu.2023.1096220)
Supplement: Supplementary file 6 [file Table_1.doc]

| primary antibody | Fluorophore |
| --- | --- |
| CD20(abcam, ab78237)  CD21(abcam, ab75985)  CD23(abcam, ab135386)  / | Opal 480 Fluorophore(Akoya)  Opal 620 Fluorophore(Akoya)  Opal 520 Fluorophore(Akoya)  DAPI(PerkinElmer, FP1490) |

**Supplementary Table S1. Reagents used for immunostaining**
