# Supplementary material for: Follicle-like tertiary lymphoid structures: A potential biomarker for prognosis and immunotherapy response in patients with laryngeal squamous cell carcinoma
Source: Front Immunol. 2023 Jan 27;14:1096220. doi: 10.3389/fimmu.2023.1096220 (PMC9912937; doi:10.3389/fimmu.2023.1096220)
Supplement: Supplementary file 7 [file Table_2.doc]

| **Table S2. Weighted Kappa** | | | | | | |
| --- | --- | --- | --- | --- | --- | --- |
| Name | Weighting | Kappa | Z | P Value | Standard Error | 95% CI |
| HE Group  &  IF Group | Quadratic | 0.810 | 5.069 | 0.000** | 0.066 | 0.681 ~ 0.939 |
| * p<0.05 ** p<0.01 | | | | | | |
