# Supplementary material for: Follicle-like tertiary lymphoid structures: A potential biomarker for prognosis and immunotherapy response in patients with laryngeal squamous cell carcinoma
Source: Front Immunol. 2023 Jan 27;14:1096220. doi: 10.3389/fimmu.2023.1096220 (PMC9912937; doi:10.3389/fimmu.2023.1096220)
Supplement: Supplementary file 8 [file Table_3.doc]

| **Table S3.Relationship between TLS and clinicopathological parameters** | | | | | | | | | |
| --- | --- | --- | --- | --- | --- | --- | --- | --- | --- |
|  | Parameters |  | TLS(%) | | | | Total | χ2 | *p* |
|  |  |  | Non-TLS | E-TLS | PFL-TLS | SFL-TLS |  |  |  |
| Test Cohort | Age（year） | ＜60 | 2(20.00) | 12(36.36) | 14(45.16) | 12(38.71) | 40(38.10) | 2.092 | 0.554 |
|  |  | ≥60 | 8(80.00) | 21(63.64) | 17(54.84) | 19(61.29) | 65(61.90) |  |  |
|  | T stage | T1-T2 | 4(40.00) | 15(45.45) | 14(45.16) | 13(41.94) | 46(43.81) | 0.162 | 0.983 |
|  |  | T3-T4 | 6(60.00) | 18(54.55) | 17(54.84) | 18(58.06) | 59(56.19) |  |  |
|  | N stage | N0 | 7(70.00) | 27(81.82) | 25(80.65) | 22(70.97) | 81(77.14) | 1.584 | 0.663 |
|  |  | Nx | 3(30.00) | 6(18.18) | 6(19.35) | 9(29.03) | 24(22.86) |  |  |
| TCGA Cohort | Age（year） | ＜60 | 5(100.00) | 13(38.24) | 8(33.33) | 5(26.32) | 31(37.80) | 9.499 | 0.023* |
|  |  | ≥60 | 0(0.00) | 21(61.76) | 16(66.67) | 14(73.68) | 51(62.20) |  |  |
|  | T stage | T1-T2 | 0(0.00) | 5(15.15) | 4(18.18) | 1(5.56) | 10(12.82) | 2.311 | 0.510 |
|  |  | T3-T4 | 5(100.00) | 28(84.85) | 18(81.82) | 17(94.44) | 68(87.18) |  |  |
|  | N stage | N0 | 3(60.00) | 15(46.88) | 11(52.38) | 8(44.44) | 37(48.68) | 0.543 | 0.909 |
|  |  | Nx | 2(40.00) | 17(53.13) | 10(47.62) | 10(55.56) | 39(51.32) |  |  |
| *** p<0.05 ** p<0.01** | | | | | | | | | |
