# Supplementary material for: Follicle-like tertiary lymphoid structures: A potential biomarker for prognosis and immunotherapy response in patients with laryngeal squamous cell carcinoma
Source: Front Immunol. 2023 Jan 27;14:1096220. doi: 10.3389/fimmu.2023.1096220 (PMC9912937; doi:10.3389/fimmu.2023.1096220)
Supplement: Supplementary file 9 [file Table_4.doc]

| **Supplementary Table S4.Chemokine list** |
| --- |
| CCL1 CCL2 CCL3 CCL4 CCL5 CCL6 CCL7 CCL8 CCL9 CCL10 CCL11 CCL12 CCL13 CCL14 CCL15 CCL16 CCL17 CCL18 CCL19 CCL20 CCL21 CCL22 CCL23 CCL24 CCL25 CCL26 CCL27 CCL28 CXCL1 CXCL2 CXCL3 CXCL4 CXCL5 CXCL6 CXCL7 CXCL8 CXCL9 CXCL10 CXCL11 [CXCL12](https://baike.baidu.com/item/CXCL12) CXCL13 CXCL14 CXCL15 CXCL16 CXCL17 XCL1 XCL2 CX3CL1 |
